# Relationships between personality traits and disordered eating among Chinese female exercisers: the role of symptoms of exercise dependence and obsessive-compulsiveness

**DOI:** 10.1186/s40337-022-00679-7

**Published:** 2022-11-17

**Authors:** Peiying Yang, Ting Wang, Fabian Herold, Notger G. Müller, Alyx Taylor, Attila Szabo, Umberto Granziol, Brian Cook, Emilio Landolfi, Marco Solmi, Liye Zou

**Affiliations:** 1 Body-Brian-Mind Laboratory, School of Psychology, Shenzhen University, Shenzhen, 518060 China; 2 Institute of Psychiatry, Psychology and Neuroscience, King’s College London, London, UK; 3 Research Group Degenerative and Chronic Diseases, Movement, Faculty of Health Sciences, University of Potsdam, Karl-Liebknecht-Str. 24-25, 14476 Potsdam, Germany; 4 School of Rehabilitation, Sport and Psychology, AECC University College, Bournemouth, BH5 2DF UK; 5 Institute of Health Promotion and Sport Science, ELTE, Eötvös Loránd Unbiversity, Budapest, Hungary; 6 Department of General Psychology, University of Padova, Padova, Italy; 7 Saint Augustine, Florida USA; 8 Faculty of Health Sciences, School of Kinesiology, University of the Fraser Valley, Abbotsford, Canada; 9 Department of Psychiatry, University of Ottawa, Ottawa, ON Canada; 10 Department of Child and Adolescent Psychiatry, Charité, Universitätsmedizin, Berlin, Germany

**Keywords:** Exercise dependence, Obsessive–compulsive symptoms, Big Five personality traits, Disordered eating, Female exercisers

## Abstract

**Background:**

Although numerous studies have examined associations between personality traits and eating disorders in females, few studies have been conducted on female exercisers. Given the high risk of disordered eating in female exercisers, this study investigated the associations between the Big Five personality traits and disordered eating in female exercisers, and further explored the potential mediators, namely exercise dependence symptoms, and obsessive–compulsive symptoms underlying this association.

**Methods:**

A total of 295 female exercisers aged between 18 to 67 years (*M* =  22.11, *SD* =  6.65) participated in this study.

**Results:**

Negative and statistically significant correlations between conscientiousness (*r* = − 0.17, *p* < 0.01), emotional stability (*r* = − 0.27, *p* < 0.001) and agreeableness (*r* = − 0.18, *p* < 0.01) and disordered eating were observed in our sample of female exercisers. The multiple mediation analyses revealed that exercise dependence symptoms and obsessive–compulsive symptoms mediate the relationship between conscientiousness (*β* = 0.016, *CI* = [0.003, 0.031]), emotional stability (*β* = -0.012, *CI* = [− 0.028, − 0.002]), and disordered eating in female exercisers, whereas obsessive–compulsive symptoms (*β* = − 0.041, *CI* = [− 0.088, − 0.001]) but not exercise dependence symptoms are a mediator of the relationship between agreeableness and disordered eating.

**Conclusions:**

Our findings can be used to improve the screening procedures for eating disorders in female exercisers as they contribute to a better understanding of the psychological mechanisms that underlie the associations between the Big Five personality traits and disordered eating.

## Plain English summary

Only a few studies have examined the associations between personality traits and disordered eating in female exercisers which, in turn, limits our current knowledge about this research topic. Given the high risk of disordered eating in female exercisers, this study investigated the associations between the Big Five personality traits (i.e., extraversion, agreeableness, conscientiousness,  emotion stability, and openness to experience) and disordered eating in female exercisers, and further explored the potential mediators, namely exercise dependence symptoms and obsessive–compulsive symptoms underlying this association. A total of 295 female exercisers aged between 18 to 67 years participated in this study. We found that in female exercisers, the level of conscientiousness, emotional stability, and agreeableness are significantly and negatively associated with the level of disordered eating. Additionally, emotional stability and conscientiousness were related to exercise dependence and obsessive–compulsive symptoms among female exercisers and could lead to disordered eating. These findings can be used to improve screening procedures for eating disorders in female exercisers as they contribute to a better understanding of the psychological mechanism underlying the associations between the Big Five personality traits and disordered eating.

## Background

Regular exercisers are at higher risk for eating disorders (EDs), exercise dependence (EXD), and obsessive–compulsive disorders (OCD) than the general population [1–3]. Furthermore, these behavioral problems (i.e. ED, EXD, OCD) are often comorbid and closely related to each other [4–7]. Personality traits such as those proposed in the Big Five model (e.g., extraversion, agreeableness, conscientiousness, neuroticism, and openness to experience: Appendix Table 6) [8] may explain the comorbidity of these behavioral problems [9–11]. However, to date, most previous studies on this research topic have been conducted in western countries, and much less is known about the above-mentioned interactions in non-western countries such as China.

The transdiagnostic model of EDs suggests that central maintaining processes are static throughout the development and maintenance across EDs [12]. Moreover, negative health consequences and detriments in quality of life are experienced even in at-risk individuals that do not go on to develop diagnosed EDs [13]. Risk of EDs is increasing in Asian countries and is often overlooked by healthcare professionals [14]. The continuum of EDs is characterized by severe disturbances in eating behavior and body image perception affecting the individual’s health and body weight [15]. Of note, a co-occurrence of mental health problems is a common phenomenon throughout the continuum of EDs, including mood disorders (depression), anxiety disorders, and EXD [16–18]. As a result, EDs are associated with an increased mortality and higher self-injury rates and  lower quality of life [19, 20].

In this context, some scholars suggest that certain personality traits are risk factors for developing EDs [21–23]. Personality refers to a set of an individual’s psychological qualities that determine their longstanding pattern of feeling, thinking, and behaviors [24]. The relationship between personality traits and EDs symptoms has been examined using the Big Five model [25]. Specifically, high scores in neuroticism (low emotion stability) and low scores in extroversion were both correlated with the risk of developing EDs [26, 27]. However, the mechanisms driving this relationship are still not clear. Furthermore, the above-mentioned associations between personality traits and EDs symptoms were observed in western women  who are at-risk of developing EDs [26, 27]. However, it is not clear whether these findings can be readily generalized to other cohorts such as female exercisers in non-western countries (e.g., China). Given that female exercisers are at high risk for EDs [1] and personality traits are associated with a greater risk of developing EDs [9], further investigations of these associations especially in non-western countries are needed.

EXD is characterized by obsessive and compulsive exercise behaviors that negatively affect one’s personal and social life [28]. A recent review revealed that perfectionism and narcissistic personality traits were linked to EXD and traits of extroversion and openness to experience were positively associated with EXD, while the other personal characteristics such as agreeableness, emotional stability, and conscientiousness were negatively associated with EXD [10]. Additionally, there is evidence of a common comorbidity among EXD and EDs [4, 18, 29–31]. Indeed, EXD and EDs commonly coexist with a prevalence rate ranging from 29 to 80% in EDs inpatients [32, 33]. In addition, the rate of comorbidity of EXD and EDs is three and a half times higher than in dependence exercisers without EDs [18]. Based on the above-presented evidence it seems reasonable to hypothesize that EXD may mediate the relationship between personality traits and severity of disordered eating, although this assumption needs to be buttressed (or refuted) by empirical evidence (e.g., in female exercisers in China).

OCD is characterized by unwanted instructive thoughts that cause anxiety or distress and that are linked to greater possibility of developing into ritualistic behaviors to prevent or reduce distress [34].

Obsessive-compulsiveness is a significant predictor of EDs [30] and is a risk factor for developing EDs [5]. Although the association between symptoms of EXD and OCD have not been extensively investigated [6], some studies noticed group differences concerning obsessive–compulsive symptoms or obsessive–compulsive traits among individuals with different levels of EXD. For example, Gulker and colleagues observed more severe obsessive–compulsive symptoms in individuals with EXD compared to individuals unaffected by this syndrome [3]. Spano found that people with high OCD score also had high excessive exercise scores [35]. The above-presented evidence suggests that a link between obsessive–compulsive symptoms and EXD-related symptoms exists. To this end, the current study also aims to examine the hypothesis that EXD symptoms predicted obsessive–compulsive (OC) symptoms among female exercisers.

**Fig. 1:**
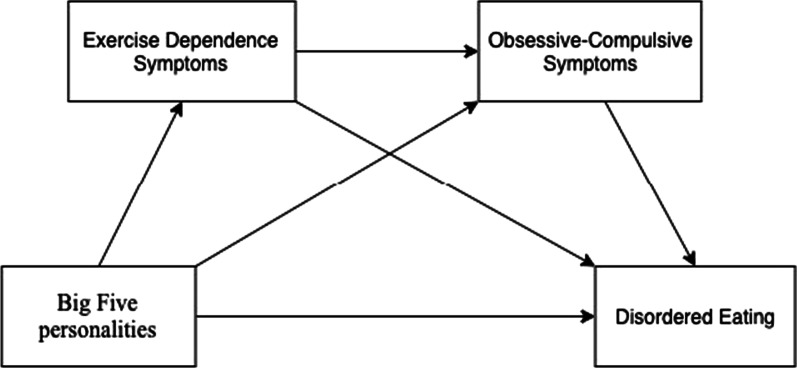
Conceptual model

In summary, the current study seeks to examine whether EXD symptoms and OC symptoms mediate the relationship between the Big Five personality traits and severity of disordered eating. More specifically, the current study aims to investigate in female exercises [1], the relationship between the Big Five personality traits and disordered eating, and [2] the mediating role of EXD symptoms and OC symptoms in the possible relationship between the Big Five personality traits and disordered eating. The conceptual multiple mediation model that arises from our hypotheses is shown in Fig. 1.

## Methods

### Participants

Using a snowball sampling system [36], female participants who habitually performed physical exercise were recruited through an online social media platform (Wechat). To be included in this study, the female exercises needed to be training  at least three times per week for a minimum of 150 min in total, for not less than six months with respect to the recruitment date. We contacted sports universities, sports teams, and exercise groups to identify eligible participants and provided interested individuals with an e-survey. The data collection started from January 1st, 2022 and ran to the March 8th, 2022. After obtaining electronic consent, participants were asked to fill out a questionnaire collecting demographic information (e.g., on gender, educational level, and physical exercise) and questionnaires assessing EXD, disordered eating, OC symptoms, and personality traits. The completion of the survey requires, on average, 10 min. Please note that two participants were deleted from the final sample due to missing and incomplete information. The study procedures are in accordance with the latest version of the Declaration of Helsinki and were approved by Ethics Committee of Shenzhen University (PN-2020–038).

### Measures

#### Exercise dependence scale-revised (EDS-R)

The 21-item EDS-R was used to assess the risk of EXD, with each item rated from 1 (“Never”) to 6 (“Always”) [37, 38]. This scale comprised 7 sub-scales (including 3 items with each sub-scale): tolerance (e.g., “I continually increase my exercise intensity to achieve the desired effects/benefits.”), withdrawal effects (e.g., “I exercise to avoid feeling irritable.”), intention effect (e.g., “I exercise longer than I intend.”), lack of control (e.g., “I am unable to reduce how long I exercise.”), time (e.g., “I spend a lot of time exercising.”), reductions in other activities (e.g., “I think about exercise when I should be concentrating on school/work.”), and continuance (e.g., “I exercise despite recurring physical problems.”). The validated Chinese version of the EDS-R has an excellent retest reliability (Cronbach's *α* = 0.95) and its scoring criteria that have been used in the current study were presented in one of our previous studies [39, 40].

#### Sick, control, one stone, fat and food (SCOFF)

The five-question SCOFF was used to identify the core features of anorexia nervosa and bulimia nervosa [41]. Participants answered either with “Yes” or “No” to the questions (e.g., “Do you worry you have lost control over how much you eat?”). One point was given for each “Yes” response, and two or more “Yes” responses (total score ≥ 2) indicate that a person is at risk for an eating disorder. The total score ranges from 0 to 5. The validated Chinese version of SCOFF [42] which has an acceptable retest reliability (Cronbach's *α* = 0.63) was used in the current study.

#### Obsessive–compulsive inventory-revised (OCI-R)

The 18-item OCI-R was used to assess severity of OC symptoms [43]. Items of the OCI-R were rated on a five-point Likert scale ranging from 0 (“Not at all”) to 5 (“Extremely”). The OCI-R consists of six factors: Washing (e.g., “I sometimes have to wash or clean myself simply because I feel contaminated.”), Obsessing (e.g., “I find it difficult to control my own thoughts.”), Hoarding (e.g., “I collect things I don’t need.”), Ordering (e.g., “I get upset if objects are not arranged properly.”), Checking (e.g., “I check things more often than necessary.”), and Mental Neutralizing (“I feel compelled to count while I am doing things.”) with 3 items of each factor. OCI-R has a cutoff score of 0–15 (mild), 16–27 (moderate), 28–72(severe) to delineating symptoms severity level [44]. The validated Chinese version of the OCI-R [45], which has been used in the current study, has an excellent retest reliability (Cronbach's *α* = 0.93).

#### The ten-item personality inventory (TIPI)

The 10-item TIPI was used to assess personality traits [46]. The items of the TIPI were rated on a seven-point Likert scale ranging from 1 (“Disagree strongly”) to 7 (“Agree strongly”). The TIPI evaluates the Big Five domains of personality traits: extroversion (e.g., “extraverted, enthusiastic”), agreeableness (e.g., “sympathetic, warm”), openness to experience (e.g., “open to new experiences, complex”), conscientiousness (e.g., “dependable, self-disciplined”), and emotional stability (e.g., “calm, emotionally stable, which is the negative opposite of neuroticism”) with two items of each personality trait. A greater score indicates a higher level of personality trait. The validated Chinese version of TIPI [47] was used in the current study.

### Statistical analysis

SPSS version 25 (IBM, NY, USA) was used for all statistical analyses. First, a descriptive analysis was conducted. Then, Pearson’s bivariate correlation was performed to examine associations between the study’s variables (each personality trait was computed by total subscale score; OC symptoms, EXD symptoms, disordered eating were computed by total score) and demographic information. According to Cohen [48], correlation coefficients of *r* = 0.10, *r* = 0.30, and *r* = 0.50 indicating a small, medium, and large effect size, respectively. In the current study, a *p*-value of < 0.05 was considered as statistically significant. The demographics being correlated with the study’s variables were controlled for further mediation analyses (i.e., age, education level). After that, based on the conceptual model that has been presented in the introduction section (see Fig. 1), the multiple mediation model was tested using Model 6 in Hayes’s PROCESS macro [49]. This mediation model allowed us to evaluate whether EXD symptoms and OC symptoms mediate the relationships between personality traits and disordered eating. An estimate of the indirect effect was tested using the standard error and 95% confidence intervals (CI) calculated from 5,000 bootstrapped samples. In accordance with the literature, a statistically significant effect was presented when the lower limit confidence interval (LLCI) and upper limit confidence interval (ULCI) did not cross zero [49–51].

## Results

### Descriptive statistics

Descriptive information of participants is shown in Table 1. The mean age of female exercisers was 22.11 years (*SD* = 6.65) with a mean BMI of 20.79 (*SD* = 3.16). Most of them undertook mixed aerobic and anaerobic exercise (67.1%), and exercised recreationally (39.7%). On average, they exercised 4.42 times a week, exercised 74.14 min every session, and had exercised for 4.82 years. Mean and *SD* for each psychological variable were calculated. Mean scores for extroversion, agreeableness, conscientiousness, emotional stability, and openness to experience were 8.82 (*SD* = 2.48), 10.67 (*SD* = 1.89), 8.67 (*SD* = 2.36), 8.80 (*SD* = 2.20), and 9.26 (*SD* = 2.06), respectively. Furthermore, their mean scores and *SD* in EXD symptoms, OC symptoms, and disordered eating were 52.70 (*SD* = 20.98), 20.39 (*SD* = 11.52), and 1.32 (*SD* = 1.37), respectively.

**Table 1:** Percentages of descriptive information and mean (*SD*) of psychological variables in our cohort of female exercisers

Variables	Female exercisers (*N* = 295)	
	*n* (%)	Mean (*SD*)
1. Education level		
Secondary/high school or professional (non-university)	15 (5.1)	
College, professional, or university training	240 (81.4)	
Post-graduate	40 (13.6)	
2. Exercise type		
Aerobic	84 (28.5)	
Anaerobic	13 (4.4)	
Mixed (both aerobic and anaerobic)	198 (67.1)	
3. Competition status		
Never compete	104 (35.3)	
Amateur/Recreational	117 (39.7)	
Athletic/Professional	74 (25.1)	
4. Exercise frequency per week		4.42 (1.88)
5. Exercise duration per session (min.)		74.17 (43.80)
6. Exercise year		4.82 (4.1)
7. Age		22.11 (6.65)
8. BMI		20.79 (3.16)
9. Big Five personality traits		
Extroversion		8.82 (2.48)
Agreeableness		10.67 (1.89)
Conscientiousness		8.67 (2.36)
Emotional Stability		8.80 (2.20)
Openness to Experience		9.26 (2.06)
10. EXD symptoms		52.70 (20.98)
11. OC symptoms		20.39 (11.52)
12. Disordered eating		1.32 (1.37)

*BMI* Body max index, *EXD* Exercise dependence, *OC* Obsessive–compulsive

### Correlation analyses

Correlations between demographic variables and psychological variables are shown in Table 2. Female exercisers with higher education levels reported fewer OC symptoms indicated by a negative correlation. In addition, we observed a positive correlation between age and a higher level of conscientiousness, emotional stability, openness to experience, but a negative correlation between age and OC symptoms, and disordered eating. BMI of female exercisers was not significantly associated with the assessed variables (i.e., personality traits, EXD symptoms, OC symptoms, and disordered eating). The r values were presented in Table 2.

**Table 2:** Bivariate correlation of demographic information and psychological variables in women exercisers (*N* = 295)

	1	2	3	4	5	6	7	8	9	10	11
1. Education level	1										
2. Age	0.35***	1									
3. BMI	0.06	− 0.02	1								
4. Extroversion	0.05	− 0.05	0.01	1							
5. Agreeableness	0.00	− 0.03	0.06	− 0.04	1						
6. Conscientiousness	0.10	0.30***	− 0.04	0.00	0.17**	1					
7. Emotional Stability	0.00	0.15*	− 0.01	− 0.08	0.33***	0.33***	1				
8. Openness to Experience	0.09	0.17**	0.10	0.21***	0.07	0.31***	0.24***	1			
9. EXD symptoms	0.02	− 0.05	− 0.02	0.22***	− 0.01	0.12*	− 0.16**	0.02	1		
10. OC symptoms	− 0.17**	− 0.18**	− 0.01	− 0.10	− 0.12*	− 0.17**	− 0.33***	− 0.25***	0.30***	1	
11. Disordered eating	− 0.03	− 0.12*	− 0.04	0.07	− 0.18**	− 0.17**	− 0.27***	− 0.07	0.13*	0.37***	1

Education level was coded as 1 = “ Secondary/high school or professional (non-university)”, 2 = “ College, professional, or university training”, and 3 = “ post-graduate”; *BMI* Body max index, *EXD* Exercise dependence, *OC* Obsessive–Compulsive

^*^p < .05, **p < .01, ***p < .001 (two-tailed)

Concerning the assessed psychological variables, we noticed that in our sample of female exercisers, a higher extroversion score is linked to more pronounced EXD symptoms, while female exercisers with higher agreeableness scores reported fewer OC symptoms and fewer disordered eating symptoms. In our sample of female exercisers higher conscientiousness and emotional stability are associated with a lower level of OC symptoms and disordered eating, but reverse effects were found with regard to EXD symptoms. Furthermore, female exercisers with higher openness to experience scores had fewer OC symptoms. In female exercisers, EXD symptoms were positively correlated with severity of OC symptoms and disordered eating. Severity of OC symptoms was positively associated with disordered eating. A more detailed overview on the r values is presented in Table 2.

### Mediation analyses

#### Mediation effect of EXD symptoms and OC symptoms in the relationship between conscientiousness and disordered eating

**Fig. 2:**
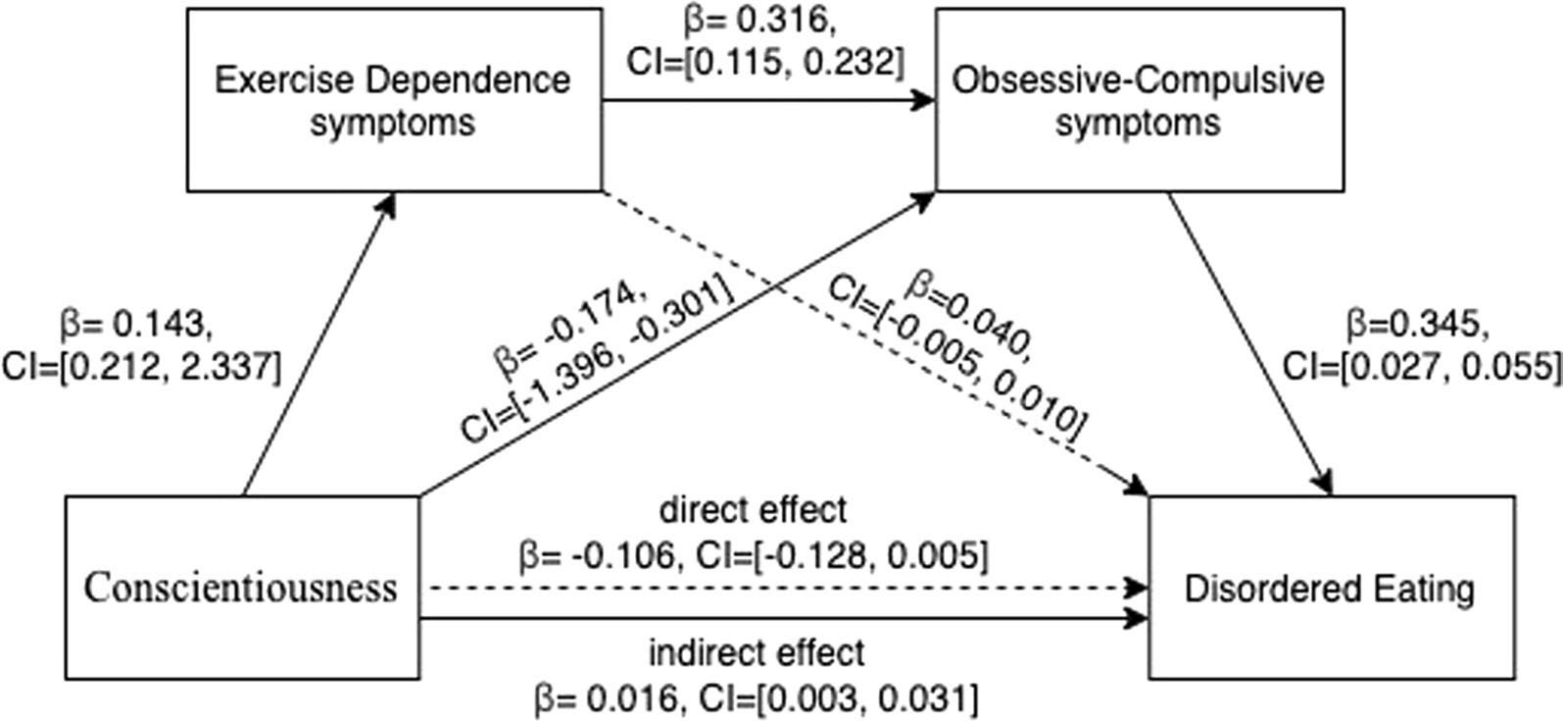
Model of effect of conscientiousness on disordered eating with EXD and OC symptoms as mediators

Given that only agreeableness, conscientiousness, and emotional stability were significantly associated with severity of disordered eating, mediation analyses were only conducted between these three personality traits and disordered eating. When age and education level were controlled, mediation analysis for conscientiousness on disordered eating showed that severity of EXD and obsessive-compulsiveness partially mediated the relationship (Fig. 2). Specifically, for the direct effect, conscientiousness was negatively but not significantly associated with disordered eating, and has significant but reverse effect on the association between the two mediators (*β* = 0.143, LLCI = 0.212–ULCI = 2.337; *β* = − 0.174, LLCI = − 1.396–ULCI = -0.301, for EXD symptoms and OC symptoms, respectively). The first mediator, EXD symptoms, was positively and significantly correlated with OC symptoms (*β* = 0.316, LLCI = 0.115–ULCI = 0.232), but not significantly correlated with disordered eating. The second mediator, OC symptoms, was positively associated with severity of disordered eating (*β* = 0.345, LLCI = 0.027–ULCI = 0.055). Concerning the indirect effect (see Table 3), although the indirect path of conscientiousness on disordered eating via EXD symptoms (path 1) was not statistically significant, the indirect path of conscientiousness on disordered eating via OC symptoms (path 2) was observed to be statistically significant (*β* = − 0.060, LLCI = − 0.115–ULCI = − 0.014). Moreover, when two mediators were included in the model, the indirect effect from conscientiousness via EXD symptoms and OC symptoms (path 3) on disordered eating was statistically significant (*β* = 0.016, LLCI = 0.003–ULCI = 0.031).

**Table 3:** Overview of the direct and indirect effects of conscientiousness on disordered eating in our cohort of female exercisers (*N* = 295)

	Standardized coefficient	*SE*	Bootstrapping	
95% *CI*				
			Lower	Upper
Direct effect	− 0.106	0.034	− 0.128	0.005
Indirect effect				
Path 1	0.006	0.009	− 0.010	0.027
Conscientiousness				
↓				
Exercise Dependence symptoms				
↓				
Disordered eating				
Path 2	− 0.060	0.026	− 0.115	− 0.014
Conscientiousness				
↓				
Obsessive–Compulsive symptoms				
↓				
Disordered eating				
Path 3	0.016	0.007	0.003	0.031
Conscientiousness				
↓				
Exercise Dependence symptoms				
↓				
Obsessive–Compulsive symptoms				
↓				
Disordered eating				
Total effect	− 0.144	0.035	− 0.153	− 0.015

Age and educational level were controlled for the model; *SE* Standard error, *CI* Confidence interval

#### Mediation effect of EXD symptoms and OC symptoms in the relationship between emotional stability and disordered eating

**Fig. 3:**
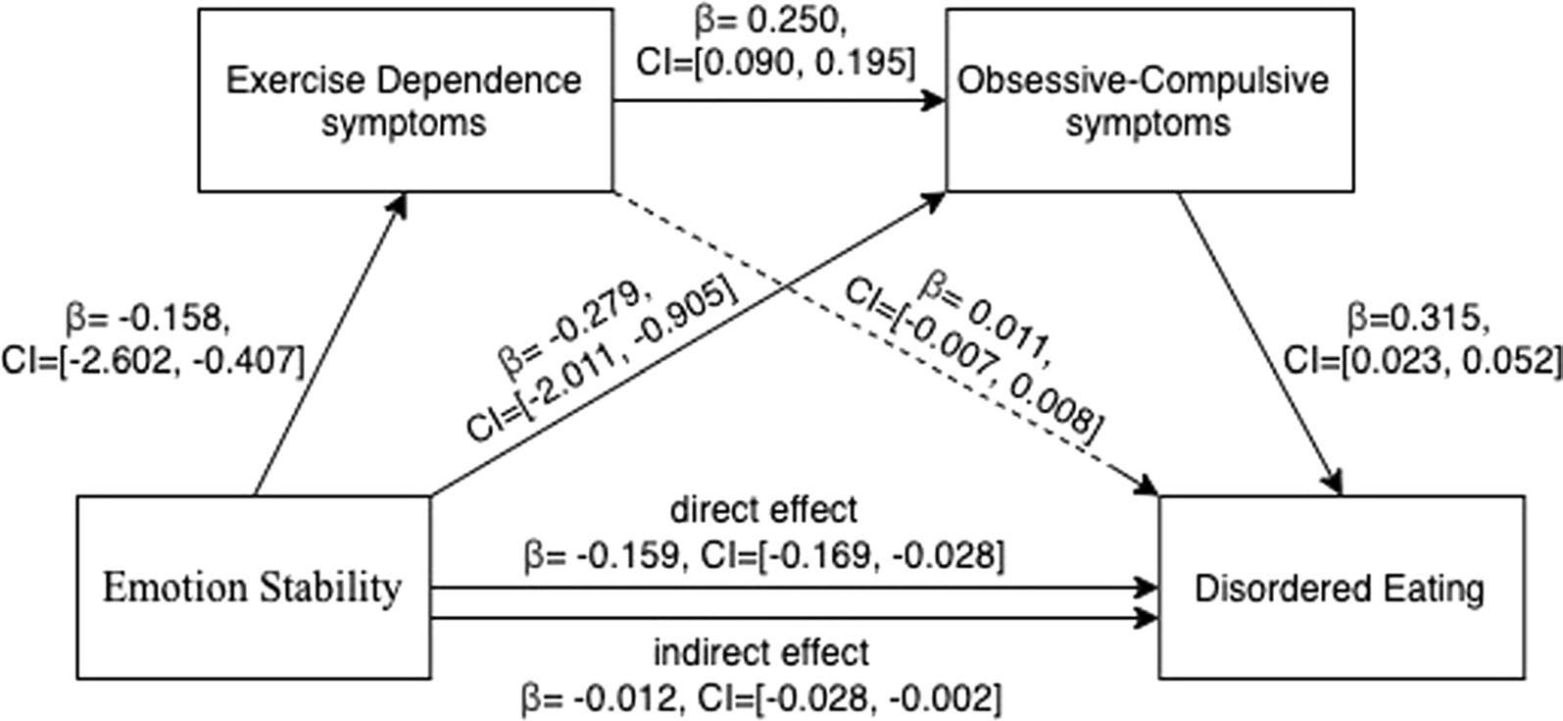
Model of effect of emotion stability on disordered eating with EXD and OC symptoms as mediators

When age and education level were controlled, mediation analysis for emotional stability on disordered eating showed that severity of symptoms of EXD and OC fully mediated this relationship (see Fig. 3). Specifically, for the direct effect, emotional stability was negatively and significantly associated with disordered eating (*β* = − 0.159, LLCI = − 0.169–ULCI = -0.028) and two mediators namely EXD symptoms (*β* = -0.158, LLCI = − 2.602–ULCI = − 0.407) and OC symptoms (*β* = − 0.279, LLCI = − 2.011–ULCI = − 0.905). The first mediator, namely EXD symptoms, was positively and significantly correlated with OC symptoms (*β* = 0.250, LLCI = 0.090–ULCI = 0.195), but not significantly correlated with disordered eating. The second mediator, namely OC symptoms, was found to be positively and significantly associated with disordered eating (*β* = 0.315, LLCI = 0.023–ULCI = 0.052). With regard to the indirect effect (Table 4), although the indirect path of emotional stability on disordered eating via EXD symptoms (path 1) was not statistically significant, the indirect path of stability on disordered eating via OC symptoms (path 2) was statistically significant (*β* = − 0.088, LLCI = − 0.146–ULCI = − 0.040). Moreover, when two mediators were included in the model, the indirect effect of emotional stability via EXD and OC symptoms (path 3) on disordered eating was statistically significant (*β* = − 0.012, LLCI = − 0.028–ULCI = − 0.002).

**Table 4:** Overview of the direct and indirect effects of Emotion stability on disordered eating in our cohort of female exercisers (*N* = 295)

	Standardized coefficient	*SE*	Bootstrapping 95% *CI*	
			Lower	Upper
Direct effect	− 0.159	0.036	− 0.169	− 0.028
Indirect effect				
Path 1	− 0.002	0.009	− 0.022	0.017
Emotion Stability				
↓				
Exercise Dependence symptoms				
↓				
Disordered eating				
Path2	− 0.088	0.027	− 0.146	− 0.040
Emotion Stability				
↓				
Obsessive–Compulsive symptoms				
↓				
Disordered eating				
Path3	− 0.012	0.007	− 0.028	− 0.002
Emotion Stability				
↓				
Exercise Dependence symptoms				
↓				
Obsessive–Compulsive symptoms				
↓				
Disordered eating				
Total effect	− 0.261	0.036	− 0.232	− 0.093

Age and educational level were controlled for the model; *SE* Standard error, *CI* Confidence interval

#### Mediation effect of EXD symptoms and OC symptoms in the relationship between agreeableness and disordered eating

**Fig. 4:**
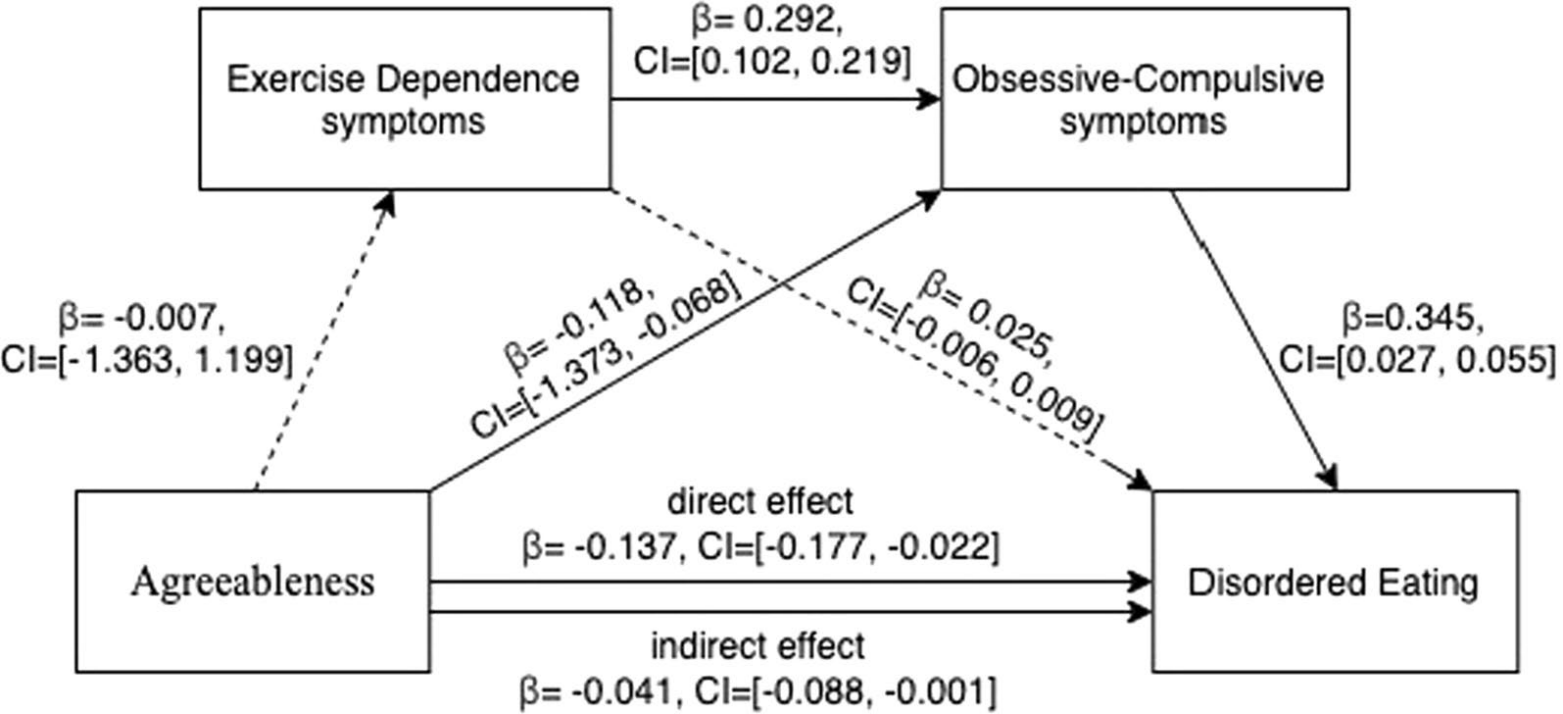
Model of effect of agreeableness on disordered eating with EXD and OC symptoms as mediators

When age and education level were controlled, the mediation analysis for agreeableness on disordered eating showed that the only OC symptoms mediated this relationship (Fig. 4). Specifically, for the direct effect, agreeableness is negatively associated with disordered eating (*β* = − 0.137, LLCI = − 0.177–ULCI = − 0.022) and OC symptoms (*β* = − 0.118, LLCI = − 1.373–ULCI = − 0.068), but not significantly associated with EXD symptoms. EXD symptoms is positively correlated with the severity of OC symptoms (*β* = 0.292, LLCI = 0.102–ULCI = 0.219), but does not show a relationship with the severity of disordered eating. OC symptoms were observed to be positively associated with disordered eating (β = 0.345, LLCI = 0.027–ULCI = 0.055). With regard to the indirect effect (Table 5), only the indirect path of agreeableness on eating disorder via OC symptoms (path 2) on disordered eating was statistically significant (*β* = − 0.041, LLCI = − 0.088–ULCI = − 0.001).

**Table 5:** Overview of direct and indirect effects of agreeableness on disordered eating in our cohort of female exercisers (*N* = 295)

	Standardized coefficient	*SE*	Bootstrapping 95% *CI*	
			Lower	Upper
Direct effect	− 0.137	0.039	− 0.177	− 0.022
Indirect effect				
Path 1	− 0.000	0.005	− 0.009	0.011
Agreeableness				
↓				
Exercise Dependence symptoms				
↓				
Disordered eating				
Path2	− 0.041	0.022	− 0.088	− 0.001
Agreeableness				
↓				
Obsessive–Compulsive symptoms				
↓				
Disordered eating				
Path3	− 0.001	0.008	− 0.018	0.013
Agreeableness				
↓				
Exercise Dependence				
↓				
Obsessive–Compulsive symptoms				
↓				
Disordered eating				
Total effect	− 0.130	0.042	− 0.212	− 0.048

Age and educational level were controlled for the model; *SE* Standard error, *CI* Confidence interval

## Discussion

The present study investigated the mediating role of symptoms of EXD and obsessive-compulsiveness in the relationship between the Big Five personality traits and disordered eating in female exercisers. Our results suggest that in female exercisers, only the personality traits (i) conscientiousness, (ii) emotional stability, and (iii) agreeableness are significantly associated with disordered eating. Furthermore, our mediation analysis revealed the relationship between conscientiousness and disordered eating was completely mediated via the path of EXD symptoms and OC symptoms, while the relationship of emotional stability and disordered eating was partly mediated via EXD symptoms and OC symptoms. Moreover, the relationship between agreeableness and disordered eating was only mediated by the severity of OC symptoms.

Concerning the relationship between personality traits and disordered eating in female exercisers, the results of the correlation analysis indicated that conscientiousness, emotional stability, and agreeableness were negatively associated with the severity of disordered eating. In contrast, extroversion and openness to experience did not show a significant association with EDs in our cohort of female exercisers. The negative relationship between conscientiousness and disordered eating is consistent with findings of previous studies observing that individuals with EDs symptoms or pathological eating behaviors showed less conscientiousness [52, 53]. The negative relationship between emotional stability and disordered eating is also in line with the observations of previous studies [26, 27]. Furthermore, this finding supports the notion that those adults, who are not able to appropriately control their emotional reactions, are more likely to exhibit EDs symptoms [54, 55]. This idea is buttressed by evidence showing that individuals with EDs suffer from difficulties regulating their emotions [56]. The negative association between agreeableness and disordered eating, which was observed in the current study, is in line with the findings of a community-based study suggesting that individuals with EDs reported significantly lower scores concerning agreeableness than individuals without EDs [57]. The latter finding might be attributable to interpersonal problems which are common among EDs patients [56]. The current study did not observe evidence for a significant association between extroversion and symptoms of EDs in our cohort of female exercisers, which is not consistent with the observations of the previous trials studying general student samples which observed a negative association [53, 58]. This may be due to the fact that exercisers have a higher level of extroversion than non-exercisers [59], and a positive association was observed between exercise dependence scores and extroversion [60], which was also  noticed in the present study. The absence of a correlation between openness to experience and disordered eating is somewhat surprising as previous studies reported group differences concerning the level of openness between individuals who suffer from EDs and healthy controls [57, 61]. However, this observation might originate from methodological differences (e.g., in analysis methods, sample size) and/or difference in the sample characteristics [54, 57, 61].

The multiple mediation model of conscientiousness on disordered eating revealed an indirect effect of conscientiousness on disordered eating via higher EXD symptoms and higher OC symptoms. This observation suggests that conscientiousness is positively related to EXD and OC symptoms among female exercisers and might lead to the occurrence of disordered eating. Although our observation is not entirely consistent with the findings of previous studies [60, 62], a positive association between conscientiousness and EXD is not that surprising as highly conscientious people have reported fewer exercise barriers [63] and higher exercise frequency [64]. The latter could be related to the fact that highly conscientious exercisers with a high level of determination and self-control also display a higher level of self-discipline and thus are more devoted to following their exercise schedule [63]. In addition, the observation of a positive relationship between disordered eating and OC symptom in present study fits the findings of previous studies [5, 65]. Several studies have demonstrated that individuals diagnosed with EDs have high comorbidity with obsessive-compulsiveness disorders [16, 66, 67]. In this context, it has been hypothesized that the co-existence of these two disorders is due to a common underlying etiology with either the two disorders representing different phases of the same disorder or with OCD being a risk factor for the development of an EDs [5]. Given the relationship between EXD, OCD, and EDs [68, 69], it seems plausible to assume that individuals with EDs who exercise excessively also show a higher level of  OC symptoms. Collectively, these findings supported the hypothesis that the effect of conscientiousness on symptoms of disordered eating is mediated via EXD and OC symptoms. Accordingly, conscientiousness is an important personality trait which can be used to screen for those at risk for EDs among female exercisers. Whether the reduction of EXD symptoms as well as OC symptoms are an effective strategy to relieve symptoms of EDs in female exercisers with high conscientious remains to be investigated in future interventional studies as such a causal conclusion is not possible with our cross-sectional study design.

The multiple mediation model of the effects of emotional stability on disordered eating demonstrated an indirect effect of emotional stability via EXD symptoms and OC symptoms in our cohort of female exercisers. This observation suggests that emotional stability is related to EXD and OC levels among female exercisers and thus might influence the occurrence of EDs, although the latter needs to be investigated in more detail in future studies. However, comparable to findings of previous studies [60, 62], emotional stability was negatively associated with EXD. As already reported in the literature emotional stability is the personality trait of the Big Five model that is most frequently associated with EXD [10]. Hausenblas and Giacobbi hypothesized that the above-mentioned relationship is related to individuals with low emotional stability who use physical exercise to cope with negative feelings, such as stress and anxiety [62]. This theoretical assumption is partly supported by the fact that individuals who suffer from EXD often report symptoms such as irritability, anxiety, depression, tension, and restlessness after a withdrawal from exercising [28]. As mentioned in the last paragraph, the positive associations between EDs symptoms and OC symptoms [5, 65] constitute risk factors for the occurrence of disordered eating [5]. The findings of our mediation analysis suggest that the relationship between emotional stability and disordered eating is influenced by EXD symptoms and OC symptoms. Thus, emotional stability seems to be an important personality trait that should be considered when female exercisers are screened for their risk of developing EDs. Moreover, our findings suggest that interventions aiming to reduce exercise dependence as well as OC symptoms may also decrease the risk of developing an ED in female exercisers with low emotional stability, although more research is necessary to provide empirical evidence for this assumption. Ecological momentary assessment studies show that dysfunctional forms of exercise, such as EXD, are used to reduce negative affect in individuals with EDs [70–72]. The results of the current study may help explain aspects of EDs etiology as they suggest that personality traits may interact with OC symptoms and EXD behavior acts upon negative affect [30]. The interaction of personality traits with presence of exercise-related compulsions (e.g., exercise dependence) and OC symptoms may provide context to explain why some individuals at-risk for EDs engage in dysfunctional exercise while others can engage in regular exercise [73] and how, and for whom, exercise can be used therapeutically [74, 75]. That is, mediators can explain why a relationship exists between independent and dependent variables [76]. Therefore, the mediating role of EXD and obsessive compulsions may distinguish individuals at-risk for EDs that will engage in dysfunctional exercise versus those that may exercise without exacerbating their eating disorder risk. Moreover, results of the current study suggest intervening on compulsions can lead to reductions in disordered eating symptom scores. Additional research is needed to evaluate these processes as part of treatment or prevention.

Considering the single mediation model of agreeableness on disordered eating, our results showed that the relationship between agreeableness and disordered eating can be explained through OC symptoms. This finding suggests that individuals with low agreeableness are more likely to exhibit OC symptoms, which, in turn, might increase the risk for EDs. Contrary to previous studies [60, 62], we did not observe a significant association between agreeableness and exercise dependence. The authors of the previous two studies explained their findings with the fact that individuals with low agreeableness are more competitive, tend to exercise longer than intended, and continue to exercise despite physical and psychological problems [60, 62]. The competition status was rather low in our sample (never compete accounted for 35.3%, and amateur/recreational accounted for 39.7%, see Table 1). This might explain the observed lack of a relationship between agreeableness with EXD. However, agreeableness was negatively related to OC symptoms, which agree with the results of Tarafder and Mukhopadhyay, who assume that low agreeableness may nurture obsessive symptoms [77]. This finding is supported by the interpersonal difficulties in OCD individuals [78, 79]. In conjunction with the potential risk factors of OCD for EDs observed in a review of longitudinal retrospective studies [5], the current study results support the notion that OC symptoms mediate the relationship between agreeableness and disordered eating. Hence, our findings suggest that agreeableness should be assessed in screenings of female exercisers when the aim is to identify individuals at a high risk for EDs. Although our cross-sectional study design did not allow to draw conclusions concerning causal relationships , our findings suggest that interventions aimed at reducing OC symptoms may be helpful to decrease the risk of developing an ED in female exercisers with low agreeableness. However, this hypothesis needs to be evaluated in future longitudinal studies.

### Contribution of the present study

The current study provides explanations for the relationship between personality traits and disordered eating in female exercisers. Results of the mediation models suggest that influence of conscientiousness and emotional stability on the risk of EDs may involve an indirect path, through the effect of EXD symptoms and OC symptoms, whereas OC symptoms only explain the influence of agreeableness on disordered eating. Although the conclusions regarding the relationship between personality traits and EDs are limited and may not be generalized to other populations, this trial is among the first studies examining the association between personality traits and disordered eating in a Chinese sample of female exercisers. Thus, the findings of the current study are a promising starting point for further investigations in this direction.

### Limitations & directions for future studies

Findings of the current study should be interpreted considering the following limitations: Firstly, this cross-sectional study design did not allow conclusions concerning the causal relationship between the study variables. Thus, experimental or longitudinal studies are needed to examine whether the observed correlations bear on causal relationships. Secondly, in the current study symptoms of disordered eating were considered, but we did not distinguish between different types of eating disorders (e.g., Anorexia nervosa / Bulimia nervosa / Binge eating disorder), which may limit the generalizability of our conclusions. Future studies should consider investigating the whether the observed relationships are influenced by different subtypes of eating disorders. Thirdly, in the current study, only Chinese female exercisers were included. Thus, it remains open whether the observed relationships could be generalized to male and non-Chinese exercisers, and thus, replication studies in other cohorts are needed. Finally, in the present study the inclusion of personality traits was operationalized by using the well-established Big Five personality model. However, given that other personality traits (e.g. perfectionism) were also identified as core characteristics among these disorders [5, 10], other personality dimensions or mechanisms underlying these relationships should be examined in future studies to allow for more robust and nuanced conclusions.

## Conclusions

In the current study, we observed in a cohort of Chinese female exercisers that EXD symptoms and OC symptoms act as mediators in the relationship between conscientiousness, emotional stability, agreeableness, and disordered eating. Our findings contribute to a better understanding of how personality traits are linked to the emergence of disordered eating in female exercisers. Given the increasing prevalence of EDs in China [80] and the vulnerability of female exercisers regarding EDs [1], these findings are of high practical relevance. Our findings can be used to improve screening procedures for EDs. The potential implications of our findings  to improve intervention strategies aiming to prevent or treat disordered eating in female exercisers need to be examined in future studies.

## Data Availability

The datasets analyzed during the current study are not publicly available because the data includes some personal information which is promised to keep confidential, but these datasets are available from the corresponding author on reasonable request.

## Appendix 1

See Table

**Table 6:** Definitions of variables used in this study

Studied variables	Definition
Big Five personality traits	
Extraversion	The dimension characterized by excitability, sociability, talkativeness, assertiveness, and high amounts of emotional expressiveness [8]
Agreeableness	The dimension includes trust, sympathy and cooperation [8]
Conscientiousness	The dimension encompasses a sense of competence and duty, and the desire to achievement and self-discipline [8]
Neuroticism (the negative opposite of Emotional Stability)	The dimension consists of a tendency to experience chronic distressing emotion such as anxiety, guilt and frustration [8]
Openness to experience (i.e. Openness)	The dimension encompasses imaginative, aesthetically sensitive, intellectual curiosity, and need for variety [8]
Exercise dependence	A phenomenon that is characterized by abnormal exercise behaviors that negatively affect one’s personal and social life [37]
Obsessive–compulsive disorder	Obsessive–compulsive disorder is characterized by unwanted instructive thoughts that caused anxiety or distress and are linked to greater possibility of developing into ritualistic behaviors to prevent or reduce distress [34]
Eating disorders	Eating disorders are characterized, for instance, by severe disturbances in eating behavior and body image perception affecting the individual’s health and body weight [16]

6.

## Appendix 2 Link of survey

Of note, this survey was developed as part of a larger, pre-registered study (preprint link: https://psyarxiv.com/64dgb/).

## Abbreviations

EDs: Eating disorders
EXD: Exercise dependence
OCD: Obsessive–compulsive disorders
